# Neonatal *NR3C1* Methylation and Social-Emotional Development at 6 and 18 Months of Age

**DOI:** 10.3389/fnbeh.2019.00014

**Published:** 2019-02-05

**Authors:** Alonzo T. Folger, Lili Ding, Hong Ji, Kimberly Yolton, Robert T. Ammerman, Judith B. Van Ginkel, Katherine Bowers

**Affiliations:** ^1^Cincinnati Children’s Hospital Medical Center, Department of Pediatrics, Division of Biostatistics and Epidemiology, University of Cincinnati College of Medicine, Cincinnati, OH, United States; ^2^Department of Anatomy, Physiology and Cell Biology, School of Veterinary Medicine, University of California, Davis, Davis, CA, United States; ^3^California National Primate Research Center, Davis, CA, United States; ^4^Cincinnati Children’s Hospital Medical Center, Department of Pediatrics, Division of General and Community Pediatrics, University of Cincinnati College of Medicine, Cincinnati, OH, United States; ^5^Cincinnati Children’s Hospital Medical Center, Department of Pediatrics, Division of Behavioral Medicine and Clinical Psychology, University of Cincinnati College of Medicine, Cincinnati, OH, United States; ^6^Cincinnati Children’s Hospital Medical Center, Department of Pediatrics, University of Cincinnati College of Medicine, Cincinnati, OH, United States

**Keywords:** *NR3C1*, glucocorticoid receptor, DNA methylation, adversity, home visiting, social-emotional development

## Abstract

The variation in childhood social-emotional development within at-risk populations may be attributed in part to epigenetic mechanisms such as DNA methylation (DNAm) that respond to environmental stressors. These mechanisms may partially underlie the degree of vulnerability (and resilience) to negative social-emotional development within adverse psychosocial environments. Extensive research supports an association between maternal adversity and offspring DNAm of the *NR3C1* gene, which encodes the glucocorticoid receptor (GR). A gap in knowledge remains regarding the relationship between *NR3C1* DNAm, measured in neonatal (1-month of age) buccal cells, and subsequent social-emotional development during infancy and early childhood. We conducted a longitudinal cohort study of *n* = 53 mother-child dyads (*n* = 30 with developmental outcomes formed the basis of current study) who were enrolled in a home visiting (HV) program. Higher mean DNAm of the *NR3C1* exon 1_F_ promoter was significantly associated with lower 6-month Ages and Stages Questionnaire: Social-Emotional (ASQ:SE) scores—more positive infant social-emotional functioning. A similar trend was observed at 18-months of age in a smaller sample (*n* = 12). The findings of this pilot study indicate that in a diverse and disadvantaged population, the level of neonatal *NR3C1* DNAm is related to later social-emotional development. Limitations and implications for future research are discussed.

## Introduction

Infant social-emotional development is foundational to later child development and behavioral health. This domain of development includes social and emotional competencies that support secure relationships and appropriate expression and regulation of emotions (Yates et al., 2008). Young children living in poverty have significantly increased risks for negative social-emotional functioning (Heberle and Carter, 2015; Steele et al., 2015). Psychosocial stressors such as maternal depression are concentrated in poverty and can disrupt infant social engagement, regulatory behaviors, and normal stress reactivity (Feldman et al., 2009). Still, differential coping capacities are observed and many disadvantaged children demonstrate positive adjustment to adverse psychosocial environments (Masten et al., 1990; Kim-Cohen et al., 2004; Rosenberg et al., 2008; Brown et al., 2012). Several factors (e.g., parental mental health, community violence) are known to mediate the effects of early disadvantage on child development (Pascoe et al., 2016), yet a need remains to elucidate the biological mechanisms that further explain vulnerability.

Epigenetic mechanisms such as DNA methylation (DNAm) may contribute to the degree of vulnerability (and resilience) to negative social-emotional development within adverse psychosocial environments. DNAm (5-methylcytosine) at CpG sites (i.e., cytosine-guanine dinucleotides) located within gene promotors can regulate gene expression. Perturbations in early environments may or may not alter DNAm levels at specific CpGs within cell types that are functionally relevant to developmental processes. Although the determinants of early development are indeed complex and multifactorial, these differences in DNAm may significantly influence developmental phenotypes—adaptive or maladaptive in nature (Lester et al., 2016; Szyf et al., 2016).

There has been extensive research that supports an association between early adversity and child DNAm of the nuclear receptor subfamily 3, group C, member 1 (*NR3C1*) gene, which encodes the glucocorticoid receptor (GR; Monk et al., 2012; Palma-Gudiel et al., 2015; Cao-Lei et al., 2017). The GR supports signaling in the hypothalamic-pituitary-adrenal (HPA) axis to regulate a stress response, including reactivity (e.g., mobilization of energy substrates) and negative feedback (Sapolsky et al., 2000; Arlt and Stewart, 2005). Animal and human studies have demonstrated that DNAm in the *NR3C1* exon 1_F_ promoter (and rat ortholog 1_7_) is associated with decreased gene expression (Weaver et al., 2007; McGowan et al., 2009). Further, *NR3C1* DNAm (placental tissue) has been associated with altered cortisol reactivity among infants (Oberlander et al., 2008; Stroud et al., 2014; Conradt et al., 2015). In response to DNAm levels at *NR3C1* regulatory regions, the quantity of GRs may differ in relevant tissues, directly affecting circulating cortisol levels and glucocorticoid sensitivity. These physiological differences may shape an infant’s regulatory behaviors and related social-emotional functioning.

Although interrogating DNAm as a molecular mediator requires more rigorous analysis of cellular models (Lappalainen and Greally, 2017), emerging evidence suggests a mechanistic role for *NR3C1* DNAm that links the early environment to profiles of neurodevelopment and behavior (Monk et al., 2012; Bromer et al., 2013; Paquette et al., 2015; Parade et al., 2016; Stroud et al., 2016). Factors such as prenatal maternal smoking and depressive symptoms have been associated with DNAm of *NR3C1* exon 1_F_ across different tissue types including placental tissue, cord blood and infant buccal cells (Oberlander et al., 2008; Braithwaite et al., 2015; Stroud et al., 2016); however, much less is known about the functional relevance of *NR3C1* DNAm to infant/child development. Existing evidence suggests that placental *NR3C1* DNAm is associated with neonatal neurobehavioral status including quality of movement, attention, and self-regulation (Bromer et al., 2013; Paquette et al., 2015; Stroud et al., 2016). Similar associations may exist between *NR3C1* DNAm in saliva specimens collected from preschool aged children and concurrent behavioral outcomes (Tyrka et al., 2015; Parade et al., 2016; Cicchetti and Handley, 2017). There is some support for using saliva/buccal tissue in epigenetic analyses as a surrogate for the target brain tissue (Smith et al., 2015). However, the potential cell type heterogeneity and a limited number of epidemiologic studies identify a need for longitudinal research devoted to this tissue type in behavioral health.

As interest in epigenetic markers (i.e., DNAm) continue to grow, there remains a lack of longitudinal studies that characterize the relationship between early (neonatal) *NR3C1* DNAm and subsequent social-emotional outcomes in diverse, disadvantaged populations. Early childhood home visiting (HV) programs are common and offer a unique opportunity to study these effects in sociodemographically high-risk populations. Therefore, the objective of this pilot study was to characterize the relationship between neonatal *NR3C1* DNAm (exon 1_F_), measured in buccal cells, and infant social-emotional development at 6 and 18 months of age within a HV population. We hypothesized that the mean *NR3C1* DNAm across 10 CpG sites in the promotor region (proximal to a known transcription binding factor) would be significantly associated with subsequent infant social-emotional functioning.

## Materials and Methods

### Participants

Participants were part of the Cincinnati Pregnancy and Infant Development (PRIDE) Study, a longitudinal cohort study of 53 at-risk mother-child dyads conducted to examine relationships between maternal prenatal factors and offspring neonatal DNAm and neurodevelopment. Eligibility criteria for the Cincinnati PRIDE Study included English-speaking mothers who were at least 18 years of age and between 12 and 35 weeks gestation at enrollment. All study participants were recruited from mothers enrolled in the Every Child Succeeds HV program in greater Cincinnati, Ohio from November 2015 to June 2016. Children in the study sample were born between December 2015 and October 2016. Every Child Succeeds home visitors were asked to approach all eligible mothers about study participation and refer those mothers who reported interest in the study. During the study time frame, 75 (30%) of an estimated 250 eligible mothers were referred to the Cincinnati PRIDE Study. Among the 75 mothers, 68 (91%) agreed to participate in the study. Following eligibility screening by study staff, 56 mothers enrolled in the study and 53 completed both study visits (described in “Procedures” section below).

Although mothers were enrolled in the HV program, participation in the Cincinnati PRIDE Study was separate, and mothers may have discharged from HV while remaining enrolled in the study. HV programs were designed to mitigate developmental risks observed in poverty by providing family supports and building positive parent-child interactions at sensitive points during early development (Sweet and Appelbaum, 2004; Adirim and Supplee, 2013). Every Child Succeeds home visits were provided to study participants under the Healthy Families America (HFA) service model (Daro and Harding, 1999). The HV program enrolls families prenatally and up to 3 months postpartum and seeks to retain them until the child is 3 years of age; however, approximately 50% of families remain active by 12-months (Folger et al., 2016). Home visits were scheduled to occur weekly or bi-weekly (depending on the point of service) during pregnancy.

### Procedures

Research assistants collected data at two study visits within the home environment. The first study visit occurred prenatally and included measurement of maternal psychological health. During the second study visit (1-month post-partum) infant buccal samples were collected for DNA isolation. Developmental screens were collected by home visitors from *n* = 30 infants who remained active in the HV program until at least 6-months of age. These data formed the basis of the current study to examine the association between neonatal DNAm at the *NR3C1* promotor (exon 1_F_) and infant 6-month social-emotional functioning. At 18-months of age, developmental screens were available and included in the analyses for a smaller subset of infants (*n* = 12). This study was carried out in accordance with the recommendations of Cincinnati Children’s Hospital Medical Center Institutional Review Board. All participants provided written informed consent in accordance with the Declaration of Helsinki. The protocol was approved by the Cincinnati Children’s Hospital Medical Center Institutional Review Board.

### Measures

Maternal and child characteristics were collected and evaluated as covariates and potential confounders. These measures included maternal race, age, education, estimated household income, smoking status, pregnancy complications (e.g., hypertension), and child’s gender and gestational age at birth (< or ≥ 37 weeks gestation). Note that household income was estimated by mother and therefore, not reported on a continuous scale; this measure did not necessarily reflect mother’s personal income. Although not collected by the Cincinnati PRIDE Study team, breastfeeding status (Yes or No) at the postpartum home visit was also gathered. Home visitors asked mothers whether they were giving breast milk to the baby at the time of the home visit, which occurred within the first month after birth.

#### Edinburgh Postnatal Depression Scale (EPDS)

The Edinburgh Postnatal Depression Scale (EPDS; Cox et al., 1987) was used to measure maternal depressive symptoms at the prenatal visit. The EPDS is a 10-item inventory used to screen for major or minor depression; higher scores are associated with elevated depressive symptoms. The total score (range 0–30) was treated as a continuous variable in the statistical models.

#### Interpersonal Support Evaluation List (ISEL-40)

The perceived interpersonal support of mothers was measured with the 40-item Interpersonal Support Evaluation List (ISEL; Cohen and Hoberman, 1983) at the prenatal study visit. A total score for overall support was derived from subscales including appraisal, tangible, self-esteem, and belonging.

#### DNA Methylation

We collected 10 buccal samples from each infant at 1-month of age, alternating between samples designated for DNA extraction (stored in lysate solution) and for cell spinning (stored in phosphate-buffered saline). Sponges were used to swab the inner cheek until saturated. Cells used for DNA extraction were collected using the DNAGenotek OGR-250 infant saliva collection kits similar to the process described by Conradt et al. (2016). DNAm measurement was performed by pyrosequencing individual bisulfite-treated DNA buccal samples subjected to PCR amplification of regions, as previously described (Ji et al., 2010, 2015; Zhang et al., 2014). Briefly, bisulfite treatment converts all non-methylated cytosine nucleotides to uracil while retaining all methylated cytosine (5-methylcytosine), allowing for the quantification of methylation through pyrosequencing after PCR amplification. DNAm of candidate CpG sites was measured by quantitative pyrosequencing using the PyroMark Q96 MD system (Qiagen) and the Pyro Q-CpG methylation software 1.0 (Qiagen). The pyrosequencing assay was validated using *Sss*I-treated human genomic DNA as a 100% methylation control and human genomic DNA amplified by GenomePlex^®^ Complete WGA kit (Sigma, St. Louis, MO, USA) as 0% methylation control. All samples were sequenced in triplicates and repeated if differed by >2%. Beta values were derived to estimate the percent methylation in each sample.

The current study focus included 10 CpG sites located in the promotor region of the *NR3C1* 1_F_ exon identified as in Braithwaite et al., 2015. The PCR and pyrosequencing primers and CpG genomic coordinates are provided in Table 1. These were sites shown in previous research to affect gene expression and have associations with prenatal maternal stressors (Oberlander et al., 2008; McGowan et al., 2009; Braithwaite et al., 2015). CpG sites 8 and 9 are consensus binding sites for the transcription factor nerve growth factor-inducible protein A (NGFI-A), suggested in human and animal studies to modulate expression of the GR (Weaver et al., 2007; Oberlander et al., 2008; McGowan et al., 2009; Braithwaite et al., 2015).

**Table 1:** Bisulfite pyrosequencing primers.

**NR3C1 1F Assay 2** chr5:143, 404, 013-143, 404, 147^†^ (strand)
PCR primer forward (5′ biotinylated)	GTTGTTATTAGTAGGGGTATTGG
PCR primer reverse	AACCACCCAATTTCTCCAATTTCTTTTC
Pyrosequencing primer (reverse)	CAACTCCCCCACTCCAAACCC
Targeted CpG sites 1–5	chr5:143, 404, 124; 143, 404, 121; 143, 404, 114; 143, 404, 099; 143, 404, 091
**NR3C1 1F Assay 1** chr5:143, 404, 011-143, 404, 097^†^ (strand)
PCR primer forward	AGTTTTAGAGTGGGTTTGGAG
PCR primer reverse (5′ biotinylated)	AAAACCACCCAATTTCTCCAATTTCTT
Pyrosequencing primer (forward)	GAGTGGGTTTGGAGT
Targeted CpG sites 6–10	chr5:143, 404, 075; 143, 404, 073; 143, 404, 063; 143, 404, 057; 143, 404, 043

*^†^Chromosomal coordinates for PCR and sequencing are based on UCSC Genome Browser Human December 2013 (GRCh38/hg38) Assembly*.

#### Cell Type Heterogeneity

We estimated the proportion of cell types in our biosamples. Cells were spun onto a glass slide, dried overnight and Hema 3-stained. Slides were then mounted and examined under microscope to estimate cell composition. Nearly 100% were buccal epithelial cells with a minimal number of leukocytes (1%–2% macrophages). Due to a uniform sample of cells, it was unnecessary to control for cell type in our statistical models.

#### Ages and Stages Questionnaire: Social-Emotional (ASQ:SE)

The Ages and Stages Questionnaire: Social-Emotional (ASQ:SE; Squires et al., 2002) was collected during routine HV service and used to measure the outcome of social-emotional functioning of children at 6 and 18-months of age. The ASQ:SE is a well-validated, parent-completed screening tool that contains items to assess infant/child competencies and problem behaviors in the dimensions of self-regulation, compliance, communication, adaptive functioning, autonomy, affect, and interaction with people. The tool has generally high internal consistency and test-rest reliability and acceptable sensitivity (range: 0.75–0.89) and specificity (range: 0.82–0.96; Squires et al., 2001). ASQ:SE items are used to identify problem behaviors and strengths and include questions at 6-months such as “When upset, can your baby calm down within a half-hour?” Home visitors trained in clinical evaluation introduced the ASQ:SE to mothers prior to administration; they were available to answer questions as mothers completed 19 and 26 questions at 6- and 18-months, respectively. We used the ASQ:SE score as a continuous outcome as in previous research (Folger et al., 2017). Higher scores are associated with poorer social-emotional functioning.

### Statistical Analysis

Descriptive analyses were conducted to examine the sociodemographic and psychosocial characteristics of the study sample (*n* = 30) with child ASQ:SE measures at 6 months. The study sample of *n* = 30 was compared to the Cincinnati PRIDE Study population of *n* = 23 mother-child dyads who did not have an ASQ:SE at 6 months. Bivariate comparisons were performed using the chi-square or Fisher’s exact chi-square tests for categorical variables and student’s *t-tests* or Wilcoxon rank-sum tests for continuous variables. Spearman correlation coefficients were calculated for percent DNAm and ASQ:SE measures.

A multivariable general linear model was constructed in which the ASQ:SE score was regressed on the measured intensity of *NR3C1* DNAm. First, DNAm was averaged across the 10 CpG sites, obviating the need to adjust for multiple comparisons in the primary study analysis. Next, DNAm beta values (i.e., interpreted as percent methylation) were transformed to M-values (log_2_ ratio of methylation percentage). The M-value was used as the main effect in the model because of the desired statistical properties over beta values (Du et al., 2010). Last, multiple variables were evaluated for inclusion in the model as potential confounding factors: maternal age, race, education, and household income; maternal prenatal depressive symptoms (EPDS); maternal interpersonal supports (ISEL-40); maternal smoking status; maternal breastfeeding status; and the quantity of prenatal home visits (i.e., dose of service). Variables were retained in the final model if statistically significant (*p*-value < 0.05) and/or there was* a priori* or observed evidence of confounding. Standardized regression coefficients were also derived from the model. We assessed multicollinearity using the variance inflation factor and condition index. There was no missing data on the parameters selected for model inclusion. An alternative multivariable tobit model was fit to account for the left-truncated ASQ:SE distribution, in which *n* = 9 children scored a zero.

In a secondary analysis, Spearman correlation coefficients were calculated to interrogate each individual CpG site. In separate multivariable models, we tested the independent associations between each CpG site and the study outcome. A false discovery rate adjustment was applied to the *p*-values generated from the individual models. All analyses were performed in SAS 9.4.

## Results

### Sample Characteristics

The primary sample of *n* = 30 mother-child dyads had similar characteristics to the full Cincinnati PRIDE Study population of *n* = 53, minimizing the potential for a selection bias (Table 2). However, the study sample differed significantly from the full study population by mean maternal age, mean prenatal home visits, and the proportion of babies born preterm (<37 weeks gestation). The *n* = 23 and *n* = 41 dyads who were missing the 6- and 18-month child ASQ:SE scores, respectively, were discharged from the HV program for reasons including no longer having time to participate, moving from service area, and excessive missed visits. Study participant characteristics included young maternal age, limited education, and living in poverty (Table 2). Over 50% of mothers were African American and 40% reported elevated prenatal depressive symptoms.

**Table 2:** Study sample characteristics by the sub-cohort with 6-month developmental screens.

	Full (*n* = 53)	6-months (*n* = 30)	*p*^f^
Maternal factors			
Age (years), mean (SD)	21.8 (3.3)	22.6 (3.9)	0.04
Race, *n* (%)			0.13
Black	33 (62.3)	16 (53.3)	
White	15 (28.3)	10 (33.3)	
Other	5 (9.4)	4 (13.3)	
Education, *n* (%)			0.88
High school or less	42 (79.3)	24 (80.0)	
Some college/ college degree	11 (20.8)	6 (20.0)	
Insurance^a^, *n* (%)			0.50
Medicaid or None	49 (96.1)	27 (93.1)	
Private	2 (3.9)	2 (6.9)	
Annual household income^a^, *n* (%)			0.27
Less than $25,000	28 (54.9)	14 (48.3)	
$25,000 or more	23 (45.1)	15 (51.7)	
Depressive symptoms^b^, *n* (%)			0.80
High	22 (41.5)	12 (40.0)	
Low-mod	31 (58.5)	18 (60.0)	
Interpersonal supports^c^, *n* (%)			0.71
High	13 (24.5)	9 (30.0)	
Low-mod	40 (75.5)	21 (70.0)	
Prenatal smoking, *n* (%)	10 (18.9)	4 (13.3)	0.24
Pregnancy complications^d^, *n* (%)	19 (35.9)	11 (36.7)	0.89
Breastfeeding^e^, *n* (%)	36 (72.0)	23 (76.7)	0.37
Prenatal home visits, med (IQR)	10 (7)	14.5 (11)	<0.01
Child Factors			
Gender, *n* (%)			0.18
Female	29 (54.7)	14 (46.7)	
Male	24 (45.3)	16 (53.3)	
Gestational age at birth, *n* (%)			0.03
<37 weeks	7 (13.2)	1 (3.3)	
≥37 weeks	46 (86.8)	29 (96.7)	
ASQ:SE 6-months, med (IQR)	-	7.5 (20)	-
*NR3C1*% DNAm, med (IQR)	2.0 (3.6)	2.0 (4.2)	0.54

*DNAm, DNA methylation; SD, standard deviation; med, median; IQR, interquartile range. ^a^Two records missing household income data; two missing insurance. ^b^Symptoms determined using the Edinburgh Postnatal Depression Scale (EPDS) total score ≥10. ^c^High social support determined using the Interpersonal Support Evaluation List-40 (ISEL-40) total score at or above the 75th percentile. ^d^Self-reported diagnoses of hypertension, gestational diabetes, anemia, and/or placenta previa. ^e^Breastfeeding/breastmilk reported at the postpartum home visit; three records missing data. ^f^*p*-value derived from comparison of *n* = 30 with a 6-month Ages and Stages Questionnaire: Social-Emotional (ASQ:SE) vs. *n* = 23 without a 6-month ASQ:SE*.

### Social-Emotional Function

#### Mean DNAm

**Figure 1:**
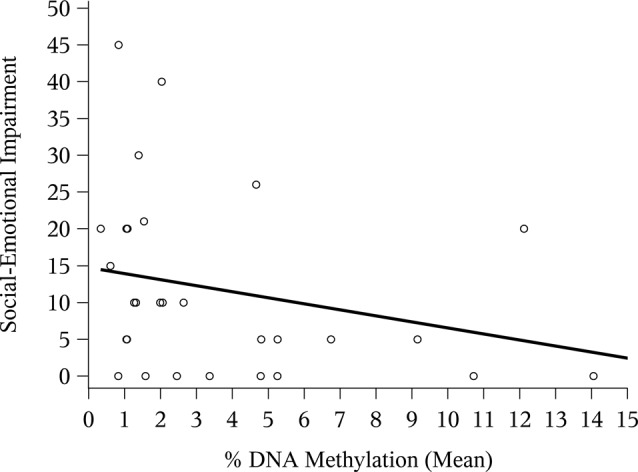
Mean *NR3C1* DNA methylation (DNAm) and 6-month Ages and Stages Questionnaire: Social-Emotional (ASQ:SE) score. Scatterplot of mean percent DNAm across CpG sites and social-emotional impairment. Higher ASQ:SE scores are associated with greater social-emotional concerns. Note that four points occupy similar coordinates, and these are represented by bolded/staggered circles.

**Figure 2:**
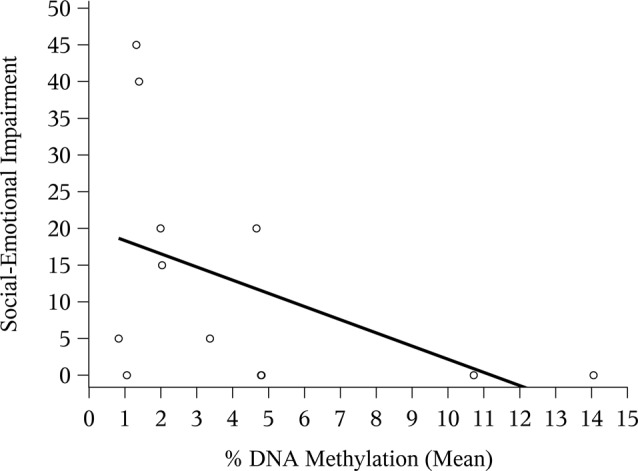
Mean *NR3C1* DNAm and 18-month ASQ:SE score. Scatterplot of mean percent DNAm across CpG sites and social-emotional impairment. Note that two points occupy similar coordinates, and these are represented by the bolded circle.

The correlation between mean *NR3C1* percent DNAm and the 6-month ASQ:SE score was negative and significant (Figure 1; Spearman correlation: −0.44, *p* = 0.02). Although not statistically significant, the correlation was again negative for the *n* = 12 children with ASQ:SE measures at 18-months of age (Figure 2; Spearman correlation coefficient: −0.52, *p* = 0.08). In an unadjusted model, mean DNAm (M-value) was a significant predictor of infant ASQ:SE score at 6-months of age [coefficient: −3.12, 95% confidence interval (CI): −5.79, −0.46]. Higher mean DNAm in the *NR3C1* promoter was associated with lower ASQ:SE scores—more positive infant social-emotional functioning. At 18 months, the unadjusted estimate followed the same trend, but was non-significant (−5.44, 95% CI: −11.90, 1.00; *p* = 0.10).

After multivariable adjustment, the effect remained statistically significant, indicating that higher/lower mean DNAm was associated with lower/higher infant ASQ:SE scores at 6-months of age (Table 3; adjusted coefficient: −3.22, 95% CI: −5.80, −0.65). A 1 standard deviation (SD) change in DNAm level was associated with a −0.41 SD change in ASQ:SE score. We included the following predictors in the multivariable model: maternal smoking status, depressive symptoms and the number of prenatal home visits received (Table 3). Maternal age, race, education, household income, ISEL-40 total score, breastfeeding status, and child gender were excluded in favor of a more parsimonious model; these measures did not improve model fit and did not appear to confound the observed DNAm effect. Note that one participant did not report estimated household income. We also excluded gestational age at birth (i.e., < or ≥ 37 weeks gestation) because this factor had little variation in the study population (Table 2). *NR3C1* DNAm accounted for 16% (semipartial η^2^: 0.158) of total variance; this was relative to 10% accounted for by both smoking and prenatal home visits and 2% by depressive symptoms. Multicollinearity was not observed (condition index: 1.6). Although the sample size was markedly reduced at 18-months of age (*n* = 12), the multivariable model was fit and suggested a larger effect than at 6-months (adjusted coefficient: −6.44, 95% CI: −13.1, 0.20; *p*-value: 0.06). The small sample size precluded the inclusion of the binary maternal smoking variable in this model.

**Table 3:** Models for the association between mean infant *NR3C1* DNAm and offspring social-emotional functioning at 6 months.

Predictors	Adjusted parameter estimates (95% CI)	*p*
Mean *NR3C1* DNAm		
M-value^a^	−3.22 (−5.80, −0.65)	0.01
M-value (tobit)^b^	−5.05 (−6.85, −3.45)	<0.01
Prenatal depressive symptoms^c^	0.38 (−0.24, 1.00)	0.23
Prenatal smoking^d^	11.81 (0.45, 23.20)	0.04
Prenatal home visiting dose^e^	−0.67 (−1.43, 0.10)	0.09

*Note: Maternal age, race, and education were non-significant and did not change the main effect (DNAm) substantially when included in model and therefore, these factors were excluded. CI, Confidence Interval. ^a^Converted beta coefficient; all other parameters were derived from the model including the M-value as main effect. ^b^Parameter estimate derived from tobit regression model; no other parameters are reported for tobit model although the same covariates were included in the model. ^c^EPDS scale; higher scores associated with elevated depressive symptoms. ^d^Self-reported ever smoking during pregnancy vs. not smoking. ^e^The quantity of home visits received during pregnancy*.

The alternative tobit regression model included the same covariates and also showed that DNAm was significant predictor at 6-month of age (adjusted coefficient: −5.05, 95% CI: −6.85, −3.85; *p* < 0.01).

#### Site-Specific DNAm

DNAm at CpG sites 6, 7 and 9 had the strongest associations with 6-month ASQ:SE scores as observed in separate multivariable models. Both of these CpG sites had correlation values and model parameter estimates that were statistically significant a *p*-value < 0.05 (Table 4). However, no sites survived FDR adjustment. CpG 9 is located at a reported NGFI-A transcription factor binding site.

**Table 4:** Associations between DNAm of *NR3C1* promotor CpG sites and offspring social-emotional functioning at 6 months.

CpG site	*R*_s_	Regression coefficient^‡^	Percent non-methylated	Range of percent methylation	IQR of percent methylation
1	−0.06	−1.63 (−5.21, 1.95)	9.4	0–9.9	0.4, 0.9
2	−0.20	−1.12 (−3.11, 0.87)	15.1	0–87.8	1.7, 4.3
3	−0.08	−0.30 (−3.77, 4.37)	1.9	0–13.6	1.7, 3.2
4	−0.29	−1.60 (−4.32, 1.13)	26.4	0–28.0	0, 2.8
5	−0.12	−1.78 (−5.27, 1.71)	17.0	0–26.8	1.7, 2.6
6	−0.38**	−1.71 (−3.35, −0.07)**	45.3	0–29.1	0, 7.7
7	−0.38**	−1.59 (−3.29, 0.11)*	47.2	0–30.0	0, 8.0
8^†^	−0.33*	−0.93 (−2.72, 0.85)	32.1	0–33.2	0, 6.3
9^†^	−0.38**	−1.92 (−3.71, −0.13)**	39.6	0–28.0	0, 5.9
10	−0.33*	−1.56 (−3.34, 0.22)*	30.2	0–27.8	0, 6.8

*p < 0.10, **p < 0.05. Note: there were no comparisons that survived FDR *p*-value adjustment. See Table 1 for chromosomal coordinates of CpG sites 1–10. R_S_: spearman correlation coefficient for relationship between Percent DNAm and ASQ:SE Score. IQR, interquartile range. ^†^Nerve growth factor-inducible protein A (NGFI-A) transcription factor binding sites. ^‡^DNAm M-values included in models; each model was adjusted for maternal prenatal depressive symptoms, smoking, and prenatal home visits.

## Discussion

Psychosocial factors in the early life environment are known to influence infant developmental trajectories. However, individual biological vulnerabilities attributed to adverse psychosocial factors remain enigmatic. Epigenetic mechanisms such as DNAm are promising targets to better understand childhood developmental risk and resilience within the context of adversity. Epigenetic changes may occur to maximize function within the anticipated environment (Blair and Raver, 2012; Shonkoff et al., 2012; Provençal and Binder, 2015); however, these psychobiological responses to adversity may be insidious and favor childhood behavioral traits that undermine healthy development. Alternatively, epigenetic responses such as increased/decreased DNAm at specific loci may favor phenotypes of resilience to early-life adversity (Van der Doelen et al., 2015). Elucidating these epigenetic effects may eventually help interventions that seek to optimize social-emotional development in the context of early adversity. Our findings indicated that within the context of psychosocial adversity the level of DNAm at *NR3C1* CpG sites was associated with differential social-emotional functioning during infancy and into early childhood.

To our knowledge, this is the first study to examine the association between neonatal *NR3C1* DNAm of buccal cells and subsequent child social-emotional development at two time points during early childhood. Further, this study uniquely examined these relationships within a diverse and disadvantaged population enrolled in a widely-disseminated prevention model. Nearly one-quarter (24%) of low income young children exhibit symptoms of social-emotional problems (Brown et al., 2012), and the negative effects of poverty are directly observed in the developing brain (Hair et al., 2015). Negative social-emotional functioning during early childhood is associated with decreased wellness in young adults across multiple domains including education, employment, and mental health (Jones et al., 2015). Early life *NR3C1* DNAm, as suggested by this work and previous research (Parade et al., 2016), may be an early and objective marker of psychosocial exposures that have significantly increased a child’s risk for negative social-emotional development and behavior. As research evolves, DNAm markers may help discern childhood phenotypes of resilience relative to those characteristic of toxic stress.

The effects we observed were the strongest at CpG sites 6, 7 and 9. CpG site 9 is located at the NGFI-A transcription factor binding site. This suggests that these sites may be integral to transcriptional regulation and impact gene expression with a direct impact on GRs and related signaling in the HPA axis. Lower *NR3C1* DNAm may support phenotypes that have increased GC sensitivity, and therefore, specific developmental vulnerabilities (Yehuda et al., 2005).

Although this research provides further support for the role of *NR3C1* DNAm in early development, the direction of our findings contradicts that of effects reported in other studies. We reported that higher mean DNAm was associated with more optimal social-emotional functioning after adjusting for factors that can impact development and potentially DNAm. However, previous studies have shown maternal prenatal adversity associated with increased *NR3C1* DNAm in varied tissue types (Oberlander et al., 2008; Braithwaite et al., 2015). Further, the postmortem brains of individual with histories of maltreatment also showed increased *NR3C1* DNAm (McGowan et al., 2009). Profiles of increased placental *NR3C1* DNAm also have been linked to increased infant cortisol reactivity (Conradt et al., 2015) and poorer infant neurodevelopment (Paquette et al., 2015). Relatedly, a study focused on child saliva specimens revealed increased *NR3C1* DNAm associated with increased internalizing (but not externalizing) behaviors in preschool (Parade et al., 2016).

Despite the aforementioned differences in effects and varied tissue types, our findings align with studies that found higher *NR3C1* DNAm of placental tissue associated with greater infant self-regulation (Conradt et al., 2015; Stroud et al., 2016). These effects were further supported by findings that suggested increased *NR3C1* DNAm of placental tissue was associated with more positive infant habituation (i.e., ability to adapt to environment), stress abstinence (i.e., regulation of physiologic and behavioral functioning), and quality of movement (Bromer et al., 2013). Although tissue specificity of *NR3C1* DNAm likely exists, both placental tissue and our target tissue, buccal epithelial cells (or saliva), may represent key developmental phenotypes and have utility as biomarkers (Armstrong et al., 2014). In a recent study of infant buccal epithelial cells, preterm infants with more medical morbidities had lower *NR3C1* DNAm, and the authors speculated DNAm as potentially promoting adaptive programming (Giarraputo et al., 2017). Further, in a study of mothers with posttraumatic stress disorder (PTSD), lower *NR3C1* DNAm in saliva was associated with greater symptom severity and parenting stress; the authors posited that *NR3C1* DNAm effect direction could be dependent on how exposures are individually “processed,” reflecting the importance of context (Schechter et al., 2015).

The contrast of effects underscores the complexity of these mechanisms and the likely importance of population context in interpreting epigenetic responses. Increased DNAm may reflect a compensatory response to prenatal adversity among some infants who have parents enrolled in a HV program. The high prevalence of poverty and interpersonal trauma (Folger et al., 2017) in our HV study population may suggest sub-groups in which *NR3C1* DNAm (CpGs 6 and 9) is protective of social-emotional health, although elucidation of these hypothesized mediated relationships requires a larger-scale study with adequate exposure variation. It could be that different psychosocial contexts—positive and negative—can result in different DNAm patterns following exposure to adversity, and therefore, have different clinical meanings. For example, Conradt et al., 2016 found that maternal parenting sensitivity moderated the relationship between maternal depressive symptoms and *NR3C1* DNAm. This suggests that although *NR3C1* DNAm is a plausible mediator for early development, the effect direction is conditional on parenting and environmental contexts, e.g., HV programs that build parental nurturance. Also important to consider are the conclusions from Bromer et al. (2013) that DNAm may be protective in one domain of development/behavior, while compromising other domains. It is also possible that the dynamic nature of DNAm during early childhood (i.e., change over time) must be measured to fully appreciate risk phenotypes (Tronick and Hunter, 2016; Parent et al., 2017).

This study has several strengths including leveraging a population of diverse, low-income mother-child dyads enrolled in a widely-implemented prevention program; the longitudinal collection of measures including prenatal adversity, neonatal DNAm and child development at 6 and 18 months of age; and a homogenous cell type (buccal epithelial cells) collected for DNAm analyses. Cell type heterogeneity can be a confounder in epidemiologic studies, and therefore, measurement and adjustment of this factor is important for unbiased effects. Saliva/buccal specimens provide a convenient approach to measure DNAm, and may provide improved interpretation over other accessible tissue types (Smith et al., 2015). Although supported by scant empirical data, limited evidence also suggests a strong correlation between *NR3C1* DNAm in buccal cells and human brain tissue (Shinozaki et al., 2017). Buccal samples may also have increased stability (i.e., technical replicate agreement) compared with other peripheral tissue types (Forest et al., 2018).

### Limitations

There were also several limitations to this study. First, the sample size was small (particularly at 18-months of age), precluding a robust assessment of mediation for investigating multiple pathways. However, this study was exploratory, and studies of similar size and focus have contributed to the emerging field (Stroud et al., 2016; Yehuda et al., 2016). Nevertheless, the study should be replicated with a larger cohort. Second, although the *NR3C1* DNAm has been linked to decreased gene expression and our target region contained a known transcription factor binding site, we did not collect RNA in this study and could not quantify the relationship between DNAm and gene expression. In addition, we restricted our focus to DNAm at the exon 1_F_, while a need remains to examine other alternative first exons that may also have mediational effects (e.g., 1_D_ and 1_H_). Third, the primary outcome measure in the study derived from a screening tool collected in practice and by parent report. This measure has been used in previous research as a continuous outcome to demonstrate the intergenerational effects of adversity (Folger et al., 2017), but future studies should include developmental and behavioral assessments administered by independent observers. Fourth, we observed low mean levels of methylation at each CpG site, which has also been observed in past research. However, low (<2%) levels of DNAm can be detected through pyrosequencing, and this study was focused on associations/correlations rather than total intensities of DNAm. Further, our primary findings seem to suggest an effect that was driven primarily by non-methylated relative to methylated (i.e., >0%) CpG sites.

## Conclusion

The study findings indicate that the level of neonatal *NR3C1* DNAm was related to later social-emotional functioning, and the effect persisted after adjustment for other predictors of development (i.e., maternal smoking, depression and dose of HV service). However, additional research in larger populations is needed to replicate and further elucidate effects. As research continues in similar populations, implications will likely emerge for prevention programs including the optimal timing of service, targeted service strategies, and determining impact.

## Data Availability

The datasets generated for this study are available on request to the corresponding author.

## Author Contributions

AF and KB were responsible for conceptualization, study design, and data collection. AF wrote the initial manuscript. LD performed data analyses and contributed to reviewing/editing the manuscript (“Results” section). HJ participated in study conceptualization, interpretation and writing (methodology). KY, RA, and JV assisted in study design, interpretation of results, and review/editing the manuscript. All authors reviewed and approved the final manuscript.

## Conflict of Interest Statement

The authors declare that the research was conducted in the absence of any commercial or financial relationships that could be construed as a potential conflict of interest.
